# Combatting cyanobacteria with hydrogen peroxide: a laboratory study on the consequences for phytoplankton community and diversity

**DOI:** 10.3389/fmicb.2015.00714

**Published:** 2015-07-22

**Authors:** Erik F. J. Weenink, Veerle M. Luimstra, Jasper M. Schuurmans, Maria J. Van Herk, Petra M. Visser, Hans C. P. Matthijs

**Affiliations:** ^1^Department of Aquatic Microbiology, Institute for Biodiversity and Ecosystem Dynamics, University of AmsterdamAmsterdam, Netherlands; ^2^Wetsus, Center of Excellence for Sustainable Water TechnologyLeeuwarden, Netherlands; ^3^Department of Aquatic Ecology, Netherlands Institute of Ecology (NIOO-KNAW)Wageningen, Netherlands; ^4^Phytoplankton Expertise Center, Koeman en BijkerkHaren, Netherlands

**Keywords:** biodiversity, harmful cyanobacteria, hydrogen peroxide, lake mitigation, phytoplankton (*Planktothrix agardhii*)

## Abstract

Experiments with different phytoplankton densities in lake samples showed that a high biomass increases the rate of hydrogen peroxide (HP) degradation and decreases the effectiveness of HP in the selective suppression of dominant cyanobacteria. However, selective application of HP requires usage of low doses only, accordingly this defines the limits for use in lake mitigation. To acquire insight into the impact of HP on other phytoplankton species, we have followed the succession of three phytoplankton groups in lake samples that were treated with different concentrations of HP using a taxa-specific fluorescence emission test. This fast assay reports relatively well on coarse changes in the phytoplankton community; the measured data and the counts from microscopical analysis of the phytoplankton matched quite well. The test was used to pursue HP application in a *Planktothrix agardhii*-dominated lake sample and displayed a promising shift in the phytoplankton community in only a few weeks. From a low-diversity community, a change to a status with a significantly higher diversity and increased abundance of eukaryotic phytoplankton species was established. Experiments in which treated samples were re-inoculated with original *P. agardhii*-rich lake water demonstrated prolonged suppression of cyanobacteria, and displayed a remarkable stability of the newly developed post-HP treatment state of the phytoplankton community.

## Introduction

In recent years the nuisance of harmful algae has increased rather than decreased (Stumpf et al., 2012; Michalak et al., 2013; Ai et al., 2015; Paerl et al., 2015), not only because of eutrophication; also climate change and rising carbon dioxide concentrations in the atmosphere make cyanobacteria thrive and contribute to their dominance (Paerl and Huisman, 2008; Carey et al., 2012; Verspagen et al., 2014). Toxin-producing cyanobacteria pose a threat to water quality in freshwater systems on a worldwide scale. It is a global interest to have ecologically healthy lakes that supply sufficient clean fresh water for fisheries, irrigation, drinking water preparation, water-recreation and esthetical aspects of the visual and odor-wise appearance of surface waters. In water quality management, long-term prevention of cyanobacterial impact is a primary goal. The preferred remedy is restoration of a lake to its original pre-eutrophication state by means of nutrient reduction, but often this may take many years, or the required engineering efforts are regarded as impossible to implement in a particular local situation, or may prove too costly (Dodds et al., 2009). In invasive lake mitigation technology, diverse methods are available for the termination of harmful cyanobacterial blooms, but many are not selective, may leave traces of added chemicals behind, and often do not comply with the desirable selectivity to not overly damage lake-ecology. An example of the latter is the use of cyanocidal or cyanostatic agents like copper sulfate; conversely a range of extracted biological agents or unrefined products, such as barley or rice straw, has been used (for a review see Jancula and Marsalek, 2011). In lakes sufficiently isolated from surrounding water bodies that may otherwise supply the lake with nutrients, phosphate reduction methods like Flock-and-Lock can terminate the yearly phosphate cycle that caters the cyanobacterial dominance (Akhurst et al., 2004; Douglas et al., 2004; Conley et al., 2009; Lürling and Van Oosterhout, 2013; Lürling et al., 2014). Alternatively, artificial deep mixing (Visser et al., 1996; Huisman et al., 2004) and flushing (see Verspagen et al., 2006 and references therein) of lakes have been successfully applied in the reduction of cyanobacterial blooms.

A more recently developed fast and effective method makes use of a higher sensitivity of cyanobacteria to hydrogen peroxide, H_2_O_2_ (hereafter HP), than other eukaryotic phytoplankton (Barroin and Feuillade, 1986; Drabkova et al., 2007a,b). The HP-method for selective suppression of cyanobacteria has already proven beneficial for entire lake mitigation in our geographical temperate zone. Fast and effective intervention against the “bad” cyanobacteria in the phytoplankton accompanies the amelioration of “food chain-positive” phytoplankton taxa like green algae, diatoms and also zooplankton. In particular, the selectivity of the HP method is of interest for ecological water management. In 2009, a dense bloom of a *Planktothrix agardhii* (hereafter *Planktothrix*)-dominated recreational lake has been successfully treated with a pre-determined minimal final lake concentration of 2.3 mg·L^−1^ HP. The HP was homogeneously introduced into the entire lake water body with a specially designed water harrow device (Matthijs et al., 2012). Within a few days, both the cyanobacterial population and the microcystin concentration collapsed for 99%. Cyanobacterial biomass remained very low until 7 weeks after the treatment, which was much longer than initially expected (Matthijs et al., 2012).

The higher sensitivity to HP-exposure of cyanobacteria as compared to eukaryotic phytoplankton is based upon a marked difference in their coping strategy to reduce high light-stress. When higher plants and eukaryotic algae are exposed to high light, HP is produced biologically as a waste product of photosynthesis (Asada, 2000, 2006; Apel and Hirt, 2004). High light causes an excess of photosynthetic electron flow, stimulating chloroplasts of plants and eukaryotic algae, such as green algae and diatoms, to use oxygen as substitute electron acceptor resulting in the formation of superoxide (Mehler reaction, Mehler, 1951). This Reactive Oxygen Species (ROS) is subsequently enzymatically converted into HP by superoxide dismutase (Latifi et al., 2009). Thereupon, catalase transforms HP into water and oxygen or alternatively anti-ROS enzymes like ascorbate peroxidase reduce HP into water (Shigeoka et al., 2002). However, in cyanobacteria, presence of flavoproteins flv1p and flv3p excludes formation of superoxide: excess electrons are still donated to oxygen but water is produced directly without intermediary production of superoxide and HP (Helman et al., 2003, 2005; Allahverdiyeva et al., 2011, 2013). By consequence, oxidative stress levels are lower in cyanobacterial cells than in eukaryotic algae and diminish the demand for elevated presence of ROS-eliminating enzymes; especially ascorbate peroxidase is lacking in cyanobacteria (Passardi et al., 2007), rendering cyanobacteria more sensitive to HP than green algae and diatoms. This encouraged testing of HP as a selective cyanocide, and the expectations about its selective capacity to kill only cyanobacteria in the phytoplankton were proven correct (Drabkova et al., 2007b).

The published dose-response observations for HP versus cyanobacteria vary substantially and range from as much as 100 mg·L^−1^ (Barrington et al., 2013) to around 60 mg·L^−1^ (Gao et al., 2015) to as low as 2 mg·L^−1^ and less (Barroin and Feuillade, 1986; Drabkova et al., 2007b). Although it is obvious that the higher the concentration of HP applied, the higher the efficiency of killing cyanobacteria will be, it must be argued that in a lake treatment the dose should be as low as possible to avoid killing of non-target species. The efficacy of homogeneously dosed, relatively low concentrations of HP for suppression of cyanobacteria in lake mitigation has already been demonstrated (Matthijs et al., 2012). However, next to these accomplishments, quite some other lake systems were regarded as not suitable for HP treatment by in advance testing at the moment a treatment was considered necessary by water managing authorities. In this study, we were interested in the reasons why in some cases a permissible HP addition of ideally less than 2.5 mg·L^−1^ to maximally 5 mg·L^−1^ was not sufficient for a major relapse of photosynthetic vitality. Our current working hypothesis for a successful treatment with HP is that exposure to a minimal HP concentration of 2 mg·L^−1^ during 5 daylight hours is required to make a treatment effective. Hence, too rapid degradation of added HP in the lake water may bring down the HP concentration too soon after addition, which greatly reduces the effectiveness in the suppression of cyanobacteria. Depending on the initial HP dose and the initial phytoplankton density, the anti-oxidative capacity of algae (or bacteria that we did not account for in this study) in the phytoplankton causes HP to degrade partly or completely even before it can start killing cyanobacteria.

However, the obvious demand for more HP contrasts with the desired exclusion of detrimental effects on sensitive taxa like the zooplankter *Daphnia*, that preferably requires application of the lowest effective concentration of HP possible (Matthijs et al., 2012). A recent acute toxicity test in the laboratory indicates a LC50 of 5.6 mg·L^−1^ HP for *Daphnia carinata*, with the no observed effect concentration (NOAEC) estimated at 3 mg·L^−1^ (Reichwaldt et al., 2012). An increased mortality for *D. carinata* above 3 mg·L^−1^ HP has been found by Barrington et al. (2013). Hence, treatments optimally require that HP degradation as catalyzed by anti-ROS enzymes should be minimal. This implies that treatments are most effective in systems with low anti-ROS capacity. To be able to predict which HP concentration would suit a treatment, in advance testing for a proper dose is essential, which includes insight into the chemical and biological rates of HP degradation.

In lakes suffering cyanobacterial nuisance, biodiversity is mostly low with only one or a few species of cyanobacteria dominating the system with major impacts on zooplankton and fish populations (Sigee, 2005; Reynolds, 2006). HP treatments in Dutch lakes have shown repeatedly that a more diverse plankton community arose after the treatment and that severe cyanobacterial blooms only reoccurred in the next growing season (Matthijs et al., 2012, and unpublished results).

In this study, we have investigated the responses of a *Planktothrix*-dominated natural phytoplankton community in small scale laboratory incubation experiments. We tested the effectiveness of the HP treatment in relation to phytoplankton density, recorded phytoplankton diversity changes in a 7-week succession experiment, and finally we re-inoculated HP treated water samples with fresh cyanobacteria-dominated water to assess sustainability of the HP treatment.

## Materials and methods

### Collection of field samples

Samples were collected on September 25, 2014 from a small water body connected to a larger water system located near the laboratory of the University of Amsterdam (Science Park) at 52°.3′N/4°9′E. The water contained a high abundance of the cyanobacterium *Planktothrix agardhii*. Water was sampled with an 8 L-bucket by pulling it from 1 m below the surface to the surface and transferred to 10 L plastic containers for transportation to the laboratory where the experiments took place. On the day of sampling, the ambient water temperature was 20°C and the day time average surface irradiation was 200 μmol photons.m^−2^.s^−1^. Samples of 200 mL were placed into 500 mL-bottles and were exposed to daylight behind a South-East facing laboratory window at a constant temperature of 20°C. Inside the lab a similar sun and cloud driven light prevalence was continued as outside, the light intensity was 30% lower inside the lab. This was compensated by the thin water layer in the incubation bottles, which was substantially less deep than in the outside lake. Bottles were exposed to ambient air, and mixed daily by hand. No nutrients were added nor measured, before, during and after the experiments. We consider the ample growth of green algae and diatoms after HP treatment as clear evidence that nutrients were not limiting.

### Concentration and dilution experiment

Three dilutions of lake samples were used: in one setting, phytoplankton was pre-concentrated; in a second, original lake water was used as collected; and in a third one the phytoplankton was diluted with lake water from which phytoplankton was removed. For the latter mild centrifugation (see below) and filtration of the supernatant through 1.2 μm Whatman glass fiber filters (GF/C, CAT No. 1822-047) with vacuum aspiration were used to yield a phytoplankton-free filtrate. For concentration, aliquots of water samples were loaded in 50 mL Falcon tubes and centrifuged at 4000 rpm at room temperature in a swing out rotor for 3 min effectively, without brakes applied at the end of the run. Half of the upper volume with clear supernatant was removed and the loose phytoplankton pellets from each set of two tubes were combined, to a final volume of 50 ml again and a total of 200 mL was put into 500 mL polyethylene bottles. The resulting phytoplankton abundance in the concentrated, original lake sample, and diluted samples was estimated by PhytoPAM (Walz, Effeltrich, Germany)-assisted relative chlorophyll measurement to be 2:1:0.5. All samples of 200 mL were transferred to 500 mL plastic bottles for a time series incubation with HP at different concentrations.

### Hydrogen peroxide degradation assay

HP was diluted from a 3% drugstore stock solution to arrive at final concentrations of 0; 2.5; 5; 10; 20; 50 mg·L^−1^ in the 200 mL described above. Just before and at 1, 2, 4, and 24 h after HP addition aliquots were taken from the thoroughly mixed bottles with volume sizes adjusted to attain comparable amounts of phytoplankton on the filters and compensate for concentration and dilution, and were filtrated over 1.2 μm Whatman glass fiber filters (GF/C, CAT No. 1842-047) for preparation of a phytoplankton-free filtrate by vacuum aspiration using a Millipore 1225 Sampling Manifold (Cat. no xx2702550 Millipore Corp. Bedford MA USA). The filtrates were used for HP quantification, by mixing 100 μL with 100 μL of *p*-nitrophenyl boronic acid reagent (Sigma) in a 96-well microtiter plate according to Lu et al. (2011). HP dependent formation of di-nitrophenol (DNP) from the reagent at room temperature was complete in 30 min and the color remained stable for several hours. DNP was measured at 405 nm using a plate reader (Molecular Devices Versamax microplate reader). Alternatively, samples of 2 mL were spun down in a microfuge at 10,000 rpm, and 100 μL of the supernatant was used for the HP assay.

### Photosynthetic vitality assay by Mini-PAM

The phytoplankton retained on the filters was used for a photosynthetic vitality assay. Photosynthetic yield was estimated with a Mini-PAM instrument according to the online user manual provided by the manufacturer (Walz, Effeltrich, Germany). The instrument was equipped with a wide bore (0.8 cm) light fiber guide, which was mounted in a stopper of the Millipore filtration apparatus. Samples on filters were left on the Millipore manifold support and rubber stoppers served to establish dark-adaption for 10 min. For a measurement the rubber stopper was rapidly exchanged by the stopper in which the miniPAM probe was mounted. Photosynthetic yield estimation followed the standard pre-set instrument protocol. While collecting phytoplankton on filters, any impact of differences in sample density were corrected by using 4 mL of the concentrated sample, 8 mL of the original lake sample and 16 mL of the diluted sample. All samples were that way measured at the same amplification setting, which permitted use of the 0 mg·L^−1^ untreated control to set the corresponding photosynthetic yield as the 100% photosynthetic vitality value. Though, the Mini-PAM assay for photosynthetic vitality does not permit to discriminate between different phytoplankton species, the fluorescence emission signal of the phytoplankton on filters proved very stable and similarly the determined effective quantum yield of PSII photochemistry, which followed from F0, Fm, and Fm' after a saturating pulse, using the preset software of the instrument, as based on Schreiber (2004).

### Relative phytoplankton species prevalence by Phyto-PAM

To determine the change in the phytoplankton community composition, the relative fluorescence was measured with a Phyto-PAM Phytoplankton Analyser. Samples of the various incubations were directly introduced into polystyrene 3 mL four sides clear fluorescence cuvettes. The Phyto-PAM instrument measures reaction center fluorescence emission after subsequent excitation with four wavelengths of 665, 645, 520, and 470 nm and transforms the emitted fluorescence signals into the approximate presence of three differently pigmented algal groups: cyanobacteria, green algae, and diatoms/dinoflagellates based on preloaded excitation/emission files of representative species of each algal group. In this study we recorded and preloaded files for laboratory cultures of the cyanobacterium *Microcystis aeruginosa* (strain PCC 7806), the green alga *Chlorella pyrenoidosa*, and the diatom *Phaeodactylum tricornutum* as internal instrument references (see Supplementary Figures 1 and 2). In the PhytoPAM assay, the relative differences in biomass prevalence are reported as differences in fluorescence intensity F_0_, as described in the user manual provided by the manufacturer (c.f. Schreiber, 2004). The Phyto-PAM assay was used just before and 4 days after HP addition to estimate changes in the relative presence of the three main phytoplankton groups.

### Succession experiment

**Figure 1:**
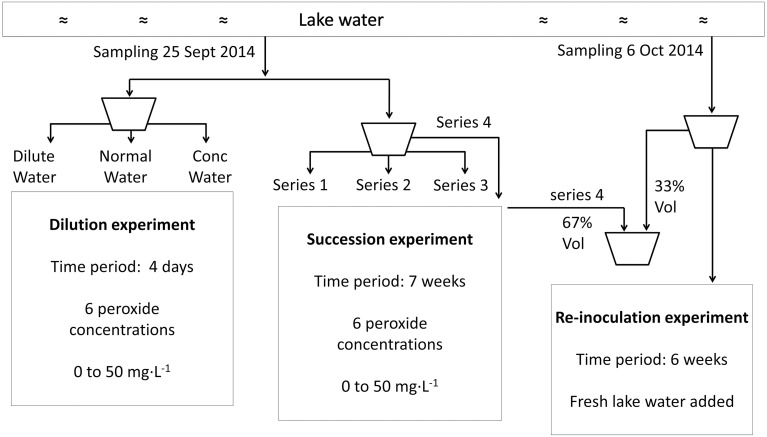
**Flow chart to illustrate the experimental set-up from sampling to incubation for the dilution experiment, succession experiment and re-inoculation experiment**.

Lake water samples (cf. Figure 1) were treated with HP to arrive at final HP concentrations of 0 (control); 2.5; 5; 10; 20; 50 mg·L^−1^, and were put at a South West facing window site in the laboratory. To determine succession in the phytoplankton community in time, the relative fluorescence was measured with a Phyto-PAM (pulse amplitude modulation) fluorometer at the time points: 0 (at start); 4; 7; 11; 15; 25; 32; and 49 days after HP addition. Since Phyto-PAM fluorescence provides no absolute numbers for phytoplankton abundance, microscopical analysis was used to confirm the established taxa discrimination and to determine actual cell numbers and biovolume. Cell counts are reported for a time point at 25 days after HP addition for 0, 2.5, 5, and 10 mg·L^−1^ (for microscopy technical description, see below).

### Re-inoculation experiment

Fresh water samples were taken on October 6 2014 to re-inoculate the samples that were treated with HP 7 days earlier (see “Succession experiment” above). The fresh water was mixed with HP treated samples at a ratio of 1:2 (fresh:treated). Prior to mixing, estimation of HP concentrations verified that HP was no longer detectable even in the samples with the highest added concentrations. As a measure for the relative abundance of different taxa, the relative fluorescence emission was obtained with the Phyto-PAM instrument (Walz, Effeltrich, Germany) just before the start at day 0 and at 4, 8, 18, 25, and 42 days after mixing.

Succession of the mixture was compared with the continued succession of the original treated series (to which no fresh water was added) and with succession of the untreated fresh water. Furthermore, at each measuring moment the expected fluorescence in imaginary instantly assembled mixtures was calculated to determine the expected community composition in case no biological community effects would have played a role.

### Microscopy

The phytoplankton samples were fixed with Lugol's Iodine. The phytoplankton was identified to genus level, and if possible to the species level, and counted using an inverted microscope using the Utermöhl-method (Utermöhl, 1958) adjusted to a European standard protocol (NEN-EN 15204, 2006). Biovolume was estimated by multiplying cell number and a standard volume for a single cell of that taxon. This volume is derived from the volume of a standard mathematical body which resembles the shape of the cell best (e.g., sphere, cylinder, spindle) using average cell dimensions based on commonly used books for determination (Komárek and Fott, 1983; Krammer and Lange-Bertalot, 1986–1991; Komárek and Anagnostidis, 2005; Coesel and Meesters, 2007).

### Data analysis

One-Way ANOVA analysis (test statistic F) followed by Tukey's pairwise comparison (test statistic Q) was conducted when four means were compared. A two-sample *t*-test was used for comparing two means. The Simpson–Dominance index (Krebs, 1989; Hillebrand and Sommer, 2000) was used to compare the dominance of a single species under different environmental conditions or similar habitats and ranges from 0, where all taxa are equally present to 1, where one taxon dominates the community completely:

(1) $$D=\sum \left({\left(\frac{ni}{N}\right)}^{2}\right)$$

Where *D* = Simpson − Dominance index (hereafter called the Dominance index), *n*_*i*_ = number of cells of each taxon (the ith taxon), and *N* = total number of cells from the sample.

The Shannon–Wiener diversity index (Shannon and Weaver, 1949), which is a function of taxa richness and evenness, was used to measure phytoplankton diversity. The index is maximal when all taxa are equally abundant in a community:

(2) $$H^{\prime }=-\sum \left[\left(\frac{ni}{N}\right)\times ln\left(\frac{ni}{N}\right)\right]$$

Where: *H*′ = Shannon diversity index, *n*_*i*_ = number of cells of each taxon (the ith taxon), and *N* = total number of cells from the sample.

Statistical analysis and calculating indices have been performed by PAST (Jin and Copper, 2008).

## Results

### Relation of phytoplankton abundance, HP degradation and photosynthetic vitality

**Figure 2:**
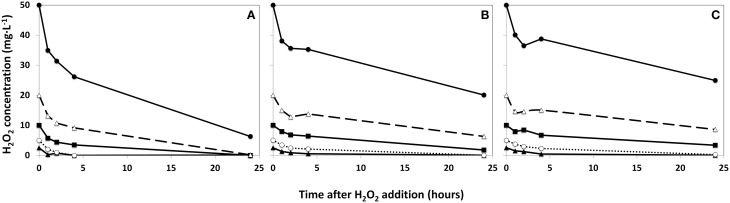
**Degradation rates of HP in lake water with a phytoplankton density of 2 × control lake water (A), water as sampled (control water, B), and diluted lake water with a phytoplankton density of 0.5 × original lake sample (C)**. After addition, the remaining HP concentration was determined at the indicated time points. 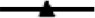 represents 2.5 mg·L^−1^; 
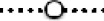
 5 mg·L^−1^; 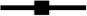 10 mg·L^−1^; 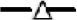 20 mg·L^−1^ and 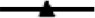 50 mg·L^−1^ HP (all are the concentrations at time zero).

Using three phytoplankton densities (2:1:0.5 ratio), the rate of degradation of added HP is shown in Figure 2. The disappearance of HP was appreciably faster in the concentrated sample (Figure 2A) than in the original lake sample (Figure 2B) and in the diluted sample (Figure 2C). As a result, no HP could be detected in samples treated with a dose of 2.5 and 5 mg·L^−1^ for concentrated water 4 h after addition, but in the diluted sample 0.4 and 4.2 mg·L^−1^ HP of the 2.5 and 5 mg·L^−1^ additions respectively were still retrieved. After 24 h, all HP was degraded in the concentrated water sample, except for the highest dose of 50 mg·L^−1^, which was still present at 6.3 mg·L^−1^ HP and of the similar start concentration the diluted water sample still contained 24.9 mg·L^−1^ HP after 24 h. The original lake sample showed no more traces of added HP below 5 mg·L^−1^ after 24 h; but of the dose of 10 mg·L^−1^ 1.8 mg·L^−1^ HP was still present (Figure 2B).

**Figure 3:**
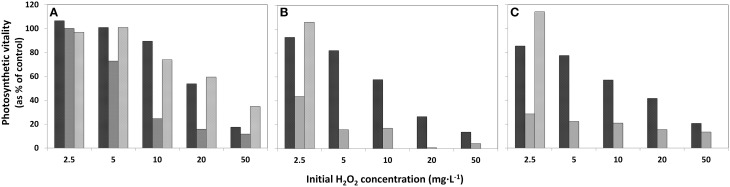
**Effects of addition of HP in different concentrations to lake water samples on the photosynthetic vitality of phytoplankton in three densities. (A)** concentrated lake water, **(B)** the original lake sample, and **(C)** diluted lake water. The bars represent photosynthetic vitality as percentage of the control, with 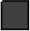 4 h after HP addition; 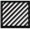 after 24 h and 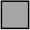 after 4 days.

The decline of the photosynthetic vitality in the phytoplankton exposed to HP was studied at 4, 24 h, and 4 days after HP addition in samples concentrated on membrane filters using the Mini-PAM instrument. Note that the decline for the concentrated sample displayed in Figure 3A is appreciably less than in the original lake sample (Figure 3B) and in the diluted sample (Figure 3C). Moreover, in the concentrated sample at all HP concentrations added, the fluorescence signal increases again after 4 days. However, for the original lake samples and diluted samples, only the lowest concentration of 2.5 mg·L^−1^ showed renewed vitality after 24 h (Figures 3B,C) and all of the higher concentrations of added HP demonstrated a further decline until below the detection limit after 4 days.

**Figure 4:**
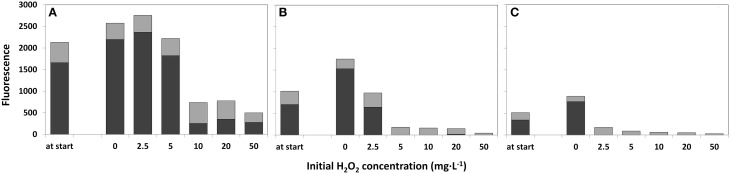
**Fluorescence emission for estimation of the relative phytoplankton abundance 4 days after treatment with a range of HP concentrations**. Concentrated lake water **(A)**, the original lake sample **(B)**, and diluted lake water **(C)** were used. The first “at start” bar represents the relative phytoplankton abundance at the start of the treatment. The relative fluorescence emission, as a measure of abundance for different taxa, was determined with a Phyto-PAM instrument. 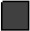 cyanobacteria; 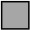 green algae plus diatoms.

The water samples that were not treated with HP (0 mg·L^−1^) demonstrated growth of cyanobacteria after 4 days as determined by the Phyto-PAM (Figure 4). The concentrated sample showed fluorescence emission of cyanobacteria for all HP additions, including the highest 50 mg·L^−1^ dose. For the original lake sample HP supplied at a dose of 5 mg·L^−1^ and higher, fluorescence signals became prominent for green algae and diatoms, and relapsed for cyanobacteria although a small signal (<15%) of cyanobacteria was detected for the 20 mg·L^−1^ test in the original lake sample water (Figure 4B). In the diluted sample, no signal from cyanobacteria was detected at any of the applied HP concentrations including the 2.5 mg·L^−1^ one. In these samples only signals from green algae and diatoms were retained. Note that the samples are studied as the original liquid suspension and the absolute height of the fluorescence is decreasing from concentrated water via original lake sample to diluted water, which is the direct consequence of the 2, 1, 0.5 phytoplankton concentration ratio's in the water sample and is not related to HP dosing.

### Species succession observed by Phyto-PAM

**Figure 5:**
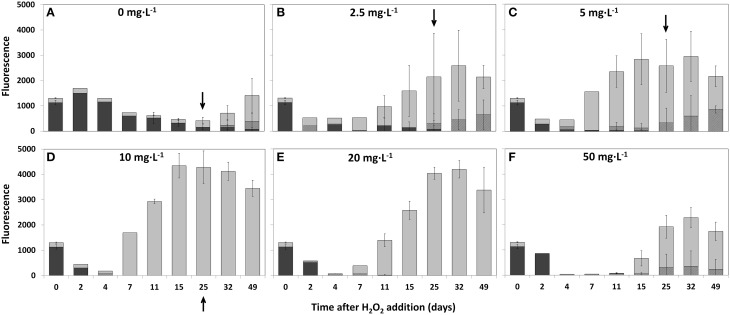
**Succession of phytoplankton groups in time determined by the relative fluorescence emission detected with the Phyto/PAM in time and with different added HP concentrations into lake water samples**. The following concentrations were used: 0 mg·L^−1^ **(A)**; 2.5 mg·L^−1^ **(B)**; 5 mg·L^−1^ **(C)**; 10 mg·L^−1^ **(D)**; 20 mg·L^−1^ **(E)**, and 50 mg·L^−1^ **(F)**. Sample size at the start of the experiment (*t* = 0) was *n* = 8. For 2, 4, and 7 days after treatment sample size was *n* = 1. All other samples sizes were *n* = 3 except for 0 mg·L^−1^ at 15, 25, 32, and 49 days which was *n* = 11 at all times. After 25 days, samples for microscopy were collected (indicated by arrows) and used for taxa discrimination and determination of cell numbers and biovolume for the 0 mg·L^−1^; 2.5 mg·L^−1^; 5 mg·L^−1^, and 10 mg·L^−1^ HP additions. 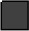 cyanobacteria; 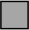 green algae and 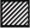 diatoms.

Changes in the composition of the phytoplankton community as a function of HP addition at time zero have been followed during 49 days (Figure 5). In the samples without added HP, fluorescence signals indicate a gradual decrease in cyanobacteria abundance after 4–7 days and a substantial increase in green algae and diatoms after 15–25 days and onwards in the untreated samples, a similar change was seen in the outside lake water (data not shown) (Figure 5A). However, in the presence of HP the succession of the dominant *Planktothrix* in the original sample to green algae and diatoms not only started earlier (already after 2–4 days at a HP dose of 2.5 mg·L^−1^), but also became more prominent (Figure 5B). The actual apparent population density as estimated by the total fluorescence signal exceeds the control after 11 days. This trend developed even more when applied HP doses increased (Figures 5C,D). Further increase of the doses to 20 and especially 50 mg·L^−1^ impeded these trends (Figures 5E,F). Diatoms became more abundant after 25 days in the 2.5 and 5 mg·L^−1^ treatments (Figures 5B,C), but were no longer found in the 10 mg·L^−1^ treatment while the signal of the green algae is still increasing (Figure 5D). At 50 mg·L^−1^, where the green algae abundance decreased, diatoms (possibly of a different peroxide resistant kind) were apparent again (Figure 5F).

The increase in fluorescence cyanobacteria signal 2 days after treatment for the different samples is remarkable. Measurements indicate no presence of cyanobacteria for the lowest dose of 2.5 mg·L^−1^ HP but the signal arose at 5 mg·L^−1^ and increased when doses increased. This aberrant increase was strongly temporal, and is possibly related to fluorescence from detached phycobilin pigments that are specifically excited in the PhytoPAM assay and started to dissappear after 4 days.

### Changes in phytoplankton composition counted by microscopy

**Figure 6:**
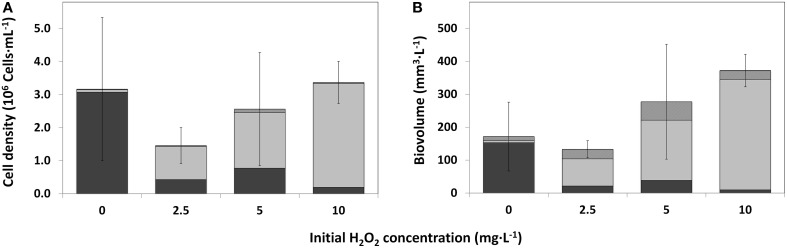
**Cell densities of the main phytoplankton groups as observed by bright field microscopy, for control water (0 mg·L^−1^) HP and treated water (2.5; 5; 10 mg·L^−1^), 25 days after HP addition. (A)** Average cell density per HP dose (*n* = 3); **(B)** Average biovolume per HP dose (*n* = 3). Error bars represent standard deviations of total cell density **(A)** and total biovolume **(B)**. 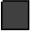 cyanobacteria; 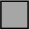 green algae; 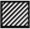 diatoms and 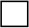 other taxa.

After 25 days, microscopy was used for comparison to the PhytoPAM data and in particular for taxa discrimination in the control (0 mg·L^−1^) and HP-treated water samples. Table 1 displays the results for the control and 2.5 mg·L^−1^ (the remaining full data including 5 and 10 mg·L^−1^ are presented in Supplementary Tables 1 and 2). All of the four HP concentrations showed considerable differences in cell densities of the main phytoplankton groups, among each other and within replicates (Figure 6A). The three control samples were nearly completely dominated by *Planktothrix*, with cell numbers ranging between 1.78·10^6^ and 5.62·10^6^ cells·mL^−1^. Two replicates of the lowest 2.5 mg·L^−1^ dose were free from cyanobacteria; however, the third replicate was still quite strongly dominated by *Planktothrix*: 1.29·10^6^ cells·mL^−1^. One replicate of the 5 mg·L^−1^ dose was free from cyanobacteria, and the other two replicates contained *Planktothrix* ranging between 0.94·10^6^ and 1.38·10^6^ cells·mL^−1^. The 10 mg·L^−1^ dose still had cyanobacteria present in two replicates, although in small numbers ranging between 0.08·10^6^ and 0.51·10^6^ cells·mL^−1^. The contribution of green algal cells in the control community was very small and ranged between 0.03·10^6^ and 0.12·10^6^ cells mL^−1^. The two replicates of the lowest 2.5 mg·L^−1^ dose without cyanobacteria, were dominated by green algae and cell numbers range between 0.89·10^6^ and 2.00·10^6^ cells·mL^−1^, where the third replicate (that still contained cyanobacteria) only showed 0.09·10^6^ cells·mL^−1^. Green algal cells in samples treated with 5 mg·L^−1^ HP were considerably more abundant and range between 0.78·10^6^ and 3.26·10^6^ cells·mL^−1^ and in samples with the 10 mg·L^−1^ dose demonstrated the highest abundances ranging between 2.50·10^6^ and 3.52·10^6^ cells·mL^−1^. Diatoms never reached high abundances but showed a slight increase in the treated samples: maximally 0.13·10^6^ cells·mL^−1^ were counted, compared to the control samples, which displayed maximally 0.04·10^6^ cells·mL^−1^.

**Table 1:** **Numbers of cells (cells·mL^−1^) of different taxa as observed by bright field microscopy**.

**TAXA**	**Control (0 mg·L^−1^ HP)**			**Treated (2.5 mg·L^−1^ HP)**		
	**1**	**2**	**3**	**1**	**2**	**3**
*Planktothrix*	1,821,818	5,620,364	1,774,545			1,287,273
CYANOBACTERIA-TOTAL	1,821,818	5,620,364	1,774,545			1,287,273
*Chlorophyta*	15,789	8525	26,885	407,139	291,228	24,918
*Closterium*			1091	2818	8000	13,455
*Coelastrum*						5246
*Crucigeniella*			2623	28,070		
*Desmodesmus*	42,105	20,984	60,328	1,471,429	365,517	43,934
*Dictyosphaerium*					203,509	
*Monoraphidium*	9211	2000	20,364	3509		3279
*Mychonastes*			7213			
*Oocystis*					3509	
*Pediastrum*				3636		
*Scenedesmus*			5246	84,211	21,053	1311
*Tetrastrum*						
GREEN ALGAE-TOTAL	125,000	61,016	212,054	3,885,833	1,557,561	174,450
*Achnanthidium*	439	4590	10,492	17,544	3509	656
*Aulacoseira*				91		
*Cymbella*				182		
*Fragilaria*			1455	818	2545	
*Navicula*				91	364	
*Nitzschia*	1754	10,182	22,545	4000	38,545	17,455
*Ulnaria*		182	2545	4182	1091	2182
DIATOMS-TOTAL	3947	25,136	59,583	30,998	84,963	37,747
OTHER-TOTAL	877		364			656

Samples from technical replicates (n = 3) of control water (0 mg·L^−1^ HP) and treated water (2.5 mg·L^−1^ HP) were collected 25 days after HP addition.

### Average cell density and biovolume by microscopical analysis

On average, total cell densities determined 25 days after treatment, were not significantly different between different treatments [Figure 6A; One-Way ANOVA, *df* = 2, *F* = 1.062, *p* = 0.418]. A clear succession was visible with increasing HP concentrations, from a *Planktothrix*-domination in control samples to a domination of green algae, even with the lowest HP concentration of 2.5 mg·L^−1^. A similar trend was observed in the biovolume shares of the main phytoplankton groups, but the total biovolume did not differ significantly between the different treatments (Figure 6B; One-Way ANOVA, *df* = 2, *F* = 3.142, *p* = 0.087). Due to different cell volumes per taxa, differences between main groups became, on average, more apparent. Diatoms contributed considerably to the community biovolume but never became dominant. The 2.5 mg·L^−1^ HP-treated samples showed on average a small decrease in total biovolume, compared to the control samples. When the doses increased, total biovolumes also tended to increase to values considerably larger than the average control samples, which is largely caused by green algal growth.

**Figure 7:**
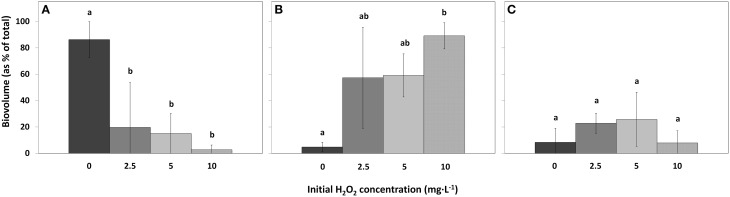
**Biovolumes of the main phytoplankton groups as percentage of the total biovolume in lake samples treated with different concentrations HP, for cyanobacteria (A), green algae (B), and diatoms (C)**. (mean ± standard deviation, *n* = 3). Significant differences (*p* < 0.05) are indicated with different letters (One-Way ANOVA analysis, Tukey *post-hoc*). The first bar 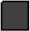 represents 0 mg·L^−1^ HP (control); second bar 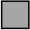 2.5 mg·L^−1^; third bar 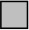 5 mg·L^−1^ and the last bar 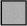 10 mg·L^−1^.

Compared to the control samples, cyanobacterial biovolumes as percentage of total biovolume, significantly decreased in the 2.5 mg·L^−1^ HP dose [*df* = 2, *Q*_(0, 2.5)_ = 5.61, *p* = 0.018]; in the 5 mg·L^−1^ dose [*df* = 2, *Q*_(0, 5)_ = 5.99, *p* = 0.012] and in the 10 mg·L^−1^ dose [*df* = 2, *Q*_(0, 10)_ = 7.04, *p* = 0.005] (Figure 7A). Biovolumes of green algae increased significantly in the 10 mg·L^−1^ HP dose compared to the control [Figure 7B; *df* = 2, *Q*_(0, 10)_ = 6.81, *p* = 0.006], where the 2.5 mg·L^−1^ dose and the 5 mg·L^−1^ dose already showed substantial and nearly significant increased compared to the control biovolume [*df* = 2, *Q*_(0, 2.5)_ = 4.24, *p* = 0.067] and [*df* = 2, *Q*_(0, 5)_ = 4.38, *p* = 0.058], respectively. Diatoms as percentage of total biovolume did not change between treatments (Figure 7C; *df* = 2, *F* = 1.574, *p* = 0.418).

### Biodiversity in HP treated samples

**Figure 8:**
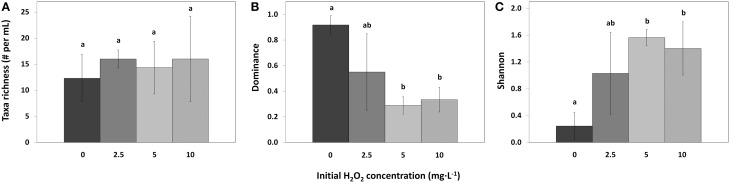
**Taxa richness (A), Simpson–Dominance index (B), and Shannon diversity index (C) of the phytoplankton in lake samples treated with different concentrations of HP (mean ± standard deviation, *n* = 3)**. Significant differences (*p* < 0.05) are indicated with different letters (One-Way ANOVA analysis, Tukey *post-hoc*). Taxa found in the samples but not counted in large numbers were not used for the analysis. The first bar 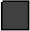 represents 0 mg·L^−1^ HP (control); second bar 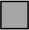 2.5 mg·L^−1^; third bar 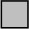 5 mg·L^−1^ and the last bar 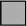 10 mg·L^−1^.

Taxa richness in the different samples was the same in control and all treatments (Figure 8A; *df* = 2, *F* = 0.32, *p* = 0.814). However, compared to the control samples, the Dominance index significantly decreased for the 5 mg·L^−1^ HP dose [Figure 8B; *df* = 2, *Q*_(0, 5)_ = 6.64, *p* = 0.007] and for the 10 mg·L^−1^ dose [*df* = 2, *Q*_(0, 10)_ = 6.17, *p* = 0.010]. The 2.5 mg·L^−1^ HP dose did not change significantly in Dominance index although a decreasing trend could be observed [Figure 8B; *df* = 2, *Q*_(0, 2.5)_ = 3.88, *p* = 0.095]. Shannon diversity index significantly increased for the 5 mg·L^−1^ dose [Figure 8C; *df* = 2, *Q*_(0, 5)_ = 5.97, *p* = 0.013] and for the 10 mg·L^−1^ dose [*df* = 2, *Q*_(0, 10)_ = 5.24, *p* = 0.025], compared to the control samples. The 2.5 mg·L^−1^ dose did not change significantly, but an increasing trend could be observed [Figure 8C; *df* = 2, *Q*_(0, 2.5)_ = 3.56, *p* = 0.132].

### Re-inoculation of HP treated samples with *Planktothrix*-rich fresh lake water

**Figure 9:**
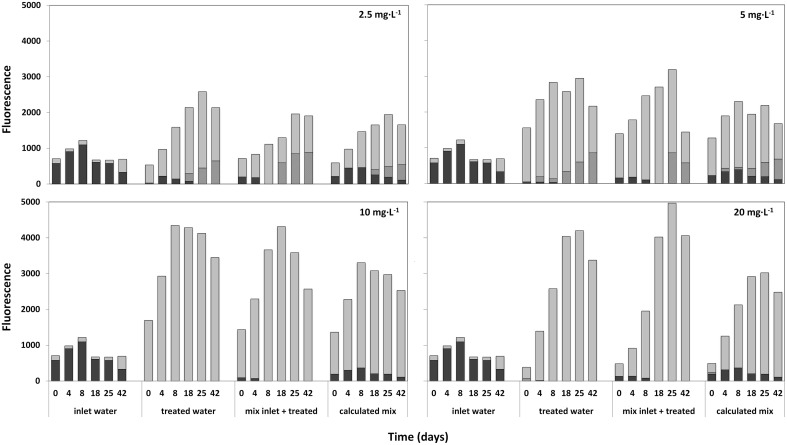
**Relative fluorescence emissions, as a measure of abundance for different taxa (determined with the Phyto-PAM) in time after mixing of fresh untreated lake water with water 7 days after treatment at a ratio of 1:2 (inlet:treated)**. Used HP concentrations of treated water were: 2.5; 5; 10; and 20 mg·L^−1^. Each figure consists of four segments with reported time series starting at day 0 (start mixing) and 4; 8; 18; 25; and 42 days after mixing. Immediately after mixing, fluorescence of the inlet water (first set of bars), the HP treated water (2nd set of bars) and the mixture (3rd set) were measured. Additionally, the calculated mean of the mixture was determined, by numerically adding the values at the time points indicated of inlet and treated water (4th set of bars). After 4 to 42 days, succession in the three samples is followed by Phyto-PAM assisted measuring of taxa-specific fluorescence for bar sets 1 and 2 and by calculating the theoretical fluorescence resulting from addition of one part fresh and two parts of treated water for each of the time points shown. Comparison of the third bar sets “mix inlet + treated” and the 4th bar sets that shows calculated outcomes if no delayed HP effects would have happened, displays how the mixture actually develops at the different HP values for treated cells. 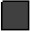 cyanobacteria; 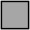 green algae and 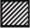 diatoms.

Lake water samples treated with 2.5; 5; 10; and 20 mg·L^−1^ HP were mixed with fresh lake water after 7 days, and the development of the composition of the phytoplankton community was followed for 42 days by Phyto-PAM (Figure 9). After an initial increase of fluorescence emission from the newly added cyanobacteria, cyanobacterial fluorescence disappeared in all treated mixtures 8 days after mixing with fresh lake water. In contrast, the cyanobacteria flourished in the inlet water. The appreciably lower prevalence of fluoresence from cyanobacteria in the third set (actually measured) relative to the fourth set (calculated result if fresh water and treated water would have been mixed at that point in time) represents prolonged cyanocidal effects in the treated samples until long after all HP had disappeared. The sustained cyanocidal effect is accompanied by an increased presence of green algae for all of the four HP concentrations tested.

## Discussion

HP changes the composition of a phytoplankton community by killing cyanobacteria in lake samples and subsequently facilitating growth of eukaryotic phytoplankton. The effectiveness depends on the biomass concentration of the phytoplankton in the original sample. HP treatment not only successfully combats the “bad” cyanobacteria in a phytoplankton population; additionally, by doing so, it greatly supports takeover by the “good” eukaryotic phytoplankton, which significantly increases biodiversity and may support efforts for ecosystem health recovery.

### How much HP has to be added for selective suppression of cyanobacteria and at which density of the phytoplankton?

In this study, we show that HP degradation is determined by the biomass density. Though use of more HP would seem an obvious solution, we advocate against such a conclusion: it is our conviction that when too much HP would be required to counteract its decrease, a treatment should not be considered. At present we recommend to use minimally 2.3 mg·L^−1^ for a treatment; this is sufficent to counteract a slight loss of HP from reductive power rendered by solutes in the water and biodegradation by the assembly of living matter (microbes, phytoplankton, zooplankton, macrophytes) in the waterbody, that is including prokaryotes other than cyanobacteria that may thrive anti-ROS capacity. The recommended HP addition value may be raised in case the reductive power of the water chemistry neutralizes a partition of typically 0.1 up to 1.2 mg·L^−1^ instantaneously, and if additionnally the biodegradation is high and reaches up to about 0.4 mg·L^−1^ HP per h, a dose of up to 5 mg·L^−1^ HP may be considered for compensation. In contrast to the concentration limits we define here, based on direct water chemistry and biochemical reduction effects, Barrington et al. (2013) recommended HP addition at a fixed ratio to the presence of chlorophyll in the water which was good for realization of phytoplankton suppression but proved harmful to zooplankton (Reichwaldt et al., 2012). The effectiveness of a certain concentration of HP added to the water to selectively kill cyanobacteria depends on the residence time of HP in the water (Matthijs et al., 2012; Burson et al., 2014). A high HP decomposition rate implies a lower residence time in the water column and thus less available time for detrimental effects on the cyanobacteria, which facilitates cyanobacteria to restore vitality and this prevents their longer-term suppression. However, we judge raising of the initial HP concentration to a concentration higher than 5 mg·L^−1^ HP as no option: even during short exposure it will risk damage to other aquatic organisms, like zooplankton, and more than 5 mg·L^−1^ measured directly after addtion is not permitted according to current local directives in The Netherlands.

High presence of anti-ROS enzymes of eukaryotic algae or the microbial community (not accounted for in this study) are most likely responsible for a high degradation rate of HP, although cyanobacteria in high enough density may to some extent contribute to peroxide degradation as well. Some cyanobacteria produce an Extracellular Polymeric Substances (EPS) layer, which is composed of polysaccharides, proteins, lipids and fatty acids (Henderson et al., 2008; Pivokonsky et al., 2014). EPS serves as a protective buffering layer around the cell and bears anti-oxidant capacity to neutralize ROS like HP (Pan, 2010; Gao et al., 2015). EPS isolated from different species of bacteria, indicated even stronger antioxidant and reactive oxygen species neutralization capacity than the typical anti-oxidant vitamin C (Chowdhury et al., 2011; Ye et al., 2012). More specifically, the cyanobacterium *Microcystis aeruginosa* demonstrates a higher HP scavenging capacity of EPS than a similar mass of pure vitamin C would realize through chemical reduction (Gao et al., 2015).

In the phytoplankton concentration and dilution experiment, we mimicked the conditions experienced during a growth season in our climate zone, with low phytoplankton abundance in early spring, and higher prevalence later in the season (Sigee, 2005; Reynolds, 2006; Palleyi et al., 2011). A simple conclusion can be drawn: treatment at an early enough moment in the season is recommended. That moment can be determined quite precisely by incubating lake samples with 0 (control), 2, 3, 5, and up to 10 mg·L^−1^ HP in the laboratory and determining the rate of HP degradation and the decline of photosynthetic vitality of the phytoplankton. A combination should be considered of a dose that will permit minimally 2 mg·L^−1^ HP to be retained until 5 h after a treatment. If that condition is realized, the decline in photosynthetic vitality will be strong enough (> 80% of the starting value as a realistic proxy) to avoid sudden recovery. And what if the right dose-effect response cannot be achieved with less than 5 mg·L^−1^? For now, referring to our experience in the Western European climate zone, we advise to start earlier in the growing season when lower phytoplankton densities still limit HP degradation. The percentage loss of the photosynthetic vitality (as tested by the mini-PAM assay) depends on the contribution of cyanobacteria in the overall phytoplankton since eukaryotic algae are not much hampered by concentrations of HP below 5 mg·L^−1^. A decrease of at least 80% of the photosynthetic value 5 h after treatment is typical for an effective treatment. This indicates a major presence of cyanobacteria in a sample, and also that HP is not broken down too rapidly as we concluded in this study from the combined assay of HP degradation and loss of photosynthetic vitality.

### Re-inoculation test for interrogation of the sustainability of lake water quality improvement

We questioned whether after a treatment, addition of an aliquot of untreated lake water with *Planktothrix* would show renewed *Planktothrix* growth. The 7 days waiting time allowed for complete degradation of the HP. In case of renewed growth, the HP method would not be contributing to resilience; if no growth resulted, the new phytoplankton state would be such that the HP treatment method is promising in terms of sustainability. In this re-inoculation experiment, where a fresh lake sample with cyanobacterial dominance was mixed with lake samples that had been treated with different HP concentrations 7 days in advance, all freshly added cyanobacteria disappeared rapidly, except in the control samples, and no sign of a return of cyanobacteria was evidenced even after 6 weeks. This is in agreement with an earlier reported HP treatment of a *Planktothrix*-invaded lake: renewed growth of *Planktothrix* remained absent until 7 weeks after the treatment. After this period the *Planktothrix* abundance increased fairly rapidly again but no more than up to a level of about 200,000 cells·mL^−1^, much lower than the 10-fold higher cell number in a nearby control lake (Matthijs et al., 2012). Eventually, it turned out that the increase in the *Planktothrix* abundance seen in the whole-lake study was caused by the intake of water from an adjacent lake to compensate for water losses in the hydrologically isolated recreation lake that was treated. The water supply was necessary to correct for evaporation after a dry and warm summer period that had lowered the absolute water depth to below the required level for waterskiing. Interestingly, the new cyanobacterial inoculum in this study did not facilitate renewed growth of cyanobacteria. In other instances of whole lake treatments, the sustainability of the HP treatment was very good as well; the suppressed cyanobacteria did not return during the remainder of the growing season, and reappeared only in the next year (unpublished results). The experiments thus showed promising resilience in which added cyanobacteria found no opportunity to regain their earlier dominance, even after addition of a substantial inoculum. Explanations for the observed sustainability of the HP method are of great interest for its actual use in lake mitigation.

So-called priority-effects, i.e., the order of arrival of colonists, can have a very strong and lasting influence on a developing population and community (Drake, 1991; Beisner et al., 2003; Schröder et al., 2005). Later immigrants can have numerical disadvantages compared to the first colonists, which may also alter the environment in a detrimental way that severely impedes colonization success for later immigrants, especially when carrying capacity is reached before later immigrants can become abundant. In a recent study, strong effects of inoculation order have been observed for multiple-strain *Microcystis*-cultures, with the priority effects being strain-specific (Van Gremberghe et al., 2009). These priority effects are likely to occur when cyanobacteria are inoculated in a dynamic and diverse community of green algae after HP application.

Several explanations for these priority-effects in the lake samples are plausible. *Planktothrix agardhii* is a species that is known to flourish under low light intensities (Mur et al., 1977). After collapse of the population, the lower plankton density may result in a higher light intensity which can hamper the growth of this species. However, the Secchi-depths in the earlier treated entire lake did not change dramatically after the treatment (Matthijs et al., 2012). Allelopathy, where green algae produce compounds that inhibit the growth of cyanobacteria, might be another explanation. In a recent study, Bittencourt-Oliveira et al. (2015) showed that the green alga *Scenedesmus acuminatus* significantly inhibited the growth of *Microcystis aeruginosa*. In our study, *Scenedesmus* is found together with other green algae like *Desmodesmus* in much larger quantities in treated samples than in the control samples (Table 1, Figures 7A,B), which could have prevented massive regrowth of surviving *Planktothrix* cells. However, cyanobacteria are particularly known for releasing biological compounds that have allelopathic properties as well (e.g., Leão et al., 2009), and this was indeed found in the same study where *M. aeruginosa* significantly inhibited the green alga *Monoraphidium convolutum* (Bittencourt-Oliveira et al., 2015). Our study similarly shows indications for allelopathic effects by *Planktothrix* on other phytoplankton: green algae only thrived when the cyanobacteria were first diminished (Figures 5B–F), whereas in the control samples the green algae remained low in numbers in the first 5 weeks after treatment (Figure 5A). Only when cyanobacteria became sufficiently low, other algae increased in abundance.

In this study, we did not investigate the role of and responses by the microbial community on changes in nutrient availability following a HP treatment. Although it can be argued that nutrient cycling may have been decreased due to a possible impact on the microbial community, the very fact that the green algae and diatoms grew vividly in our tests without any nutrients being added, convinced us that nutrient availability as such must have been ample. Regarding microbes in metabolic turnover, a few results from previous studies are available. Soon after a lake treatment with HP (Matthijs et al., 2012; unpublished observations), the presence of ammonium ions rose steeply, and also phosphate presence increased. From this we deduced active protease and nuclease/phosphatase activity, most likely provided by microorganisms in the lake water that resisted the HP treatment. We tested coliform numbers before and after a treatment: the number of live cells slightly increased temporarily, but then equilibrated to the pre-treatment level. After only a few days the extra ammonium was metabolized into nitrate, indicating that ammonium oxidizers are active. From this we conclude that microorganisms in the water that provide proteases, ammonium oxidizing capacity and phosphatases retain activity. As long as we are not well informed about HP effects on other prokaryotes, we consider that because microorganisms may reinoculate the water column from the sediment, a HP treatment should be carried out without affecting the sediment, by finishing HP addition at minimally 20 cm above the sediment. Some bacteria are also thought to play a role in scavenging HP. For example, recent experiments demonstrated that catalase-producing heterotrophic bacteria in dilute microbial communities in the surface mixed layer of the oligotrophic ocean play a vital role in protecting the cyanobacterium *Prochlorococcus* from oxidative damage (Morris et al., 2008, 2011). One of our current fields of study concerns impacts and consequences of HP for the microbial community, including the role of HP scavenging by heterotrophic bacteria, since a better ecological understanding is necessary for societal acceptance of the HP technique as an ecological safe cyanocide.

Another explanation for the longer term absence of cyanobacteria after a HP treatment may be the potential role of freshwater cyanophages that have been recognized to attribute to lake ecology and in termination of cyanobacterial blooms (Tucker and Pollard, 2005; Deng and Hayes, 2008; Steenhauer et al., 2014; Watkins et al., 2014). In marine surface waters, back ground values of naturally available HP can induce an enhanced viral production in bacterial communities (Weinbauer and Suttle, 1999). HP treatment-introduced peroxide may raise the vulnerability of cyanobacterial cells to new cyanophage infection, followed by a release of viruses. This would create a large enough cyanophage population in the water column to effectively control the cyanobacterial population.

### Phytoplankton community analysis by Phyto-PAM and microscopy

The data presented in this work introduced use of the PhytoPAM technique for coarse interrogation of the lake phytoplankton community and the acquired data were fine-tuned by classical microscopical analysis of the phytoplankton community. Direct comparison of microscopy data with those of the Phyto-PAM reveals a shortcoming of the Phyto-PAM-assisted phytoplankton composition analysis: in one or two of the treated replicates with 2.5; 5; and 10 mg·L^−1^ HP cyanobacteria were found by microscopy counting (Table 1) whilst the cyanobacterial fluorescence signal was only present at the lowest HP concentration of 2.5 mg·L^−1^ and in only one of the three replicates (Figure 5B). It appears that cyanobacterial fluorescence in a mix with eukaryotic algae, gives an underestimation of the cyanobacterial abundance and an overestimation of the other algae. This is why in microscopy remaining cyanobacteria were revealed 25 days after the HP incubation that were not detected in the Phyto-PAM assay.

Moreover, as a consequence of fluorescence emitted by free phycobilisomes, the Phyto-PAM technique is not considered suitable for monitoring immediately after HP application, especially when HP dosing was high enough to cause mass lysis of phycobilisomes. Nonetheless, after a few days, especially with a low HP treatment dose, the use of the Phyto-PAM is recommended as a fast and easy monitoring device, to distinguish between main algal groups in a coarse way. In fact, also microscopy is not well suited for fast post-treatment analysis: the integrity of some cyanobacterial cells remains visibly unaffected until quite some time after a treatment, whereas still viable green algae often show a temporary wrinkled appearance (unpublished observations).

### HP application contributes toward a desirable healthy ecosystem state

High diversity ecosystems are considered to have a higher resilience (Folke et al., 2004), where resilience can be defined as the capacity of a system to absorb disturbance and reorganize while undergoing change so as to retain essentially the same function, structure, identity, and feedbacks (Walker et al., 2004). Several studies on lake systems show that a loss of resilience, usually caused by human-induced eutrophication, often triggers a shift from a stable state with clear water rich in submerged vegetation, to an alternative stable state with turbid water, loss of biodiversity and high algal biomass (Scheffer et al., 1993, 1997, 2001). It is argued that once a system has turned into a turbid state, the threshold level for reduced nutrient load to cause the system to shift back to a clear state, is substantially lower than the threshold level which initially caused the system to become turbid. An important factor is that many cyanobacteria are not only excellent competitors under low light conditions; they are also capable, more than eukaryotic phytoplankton, of causing higher turbidity per unit of phosphorus and hence promote low light conditions (Scheffer et al., 1997). As a consequence, nutrient reduction is often unsuccessful (Sas, 1989; Scheffer et al., 1993), and additional measures like foodweb manipulation must be implemented to push a lake into a desired state. For example, biomanipulation experiments in a whole lake show that a strong reduction of fish biomass could act as a ‘shock therapy’ to turn the turbid lake back into a clear state (Meijer, 1994).

Interestingly, HP treatment when effective, causes massive killing and lysis of cyanobacterial cells resulting in temporarily increased nutrient levels. These nutrients may in turn become a food source for the remaining taxa, which are not affected by the treatment, and contribute to lake restoration with higher diversity, and food chain value. This scenario was actually displayed in our experiments, where after HP application as ‘shock therapy’ cyanobacterial dominance was rapidly replaced by a higher species diversity without any species dominance. If the application is accompanied by a reduction in internal and external nutrient loading, this new state with a diverse phytoplankton community might be established for the longer term and support lake mitigation. However, until now HP applications have been used in eutrophic systems where nutrient reduction is regarded not feasible or not yet effective, and the diverse phytoplankton community will fall back to a cyanobacteria-dominated ecosystem and require a renewed HP treatment in each new growth season.

## Concluding remarks

HP treatment established sustained suppression of cyanobacterial dominance in *Planktothrix*-dominated lake samples. The water remained perfectly suitable for phytoplankton growth; green algae and diatoms thrived. It appeared that eukaryotic phytoplankton responded favorably to the suppression of cyanobacteria and that in retrospect, the green algae gained enhanced competitiveness and restrained cyanobacteria from re-establishing in the ecosystem. This is a highly interesting observation and is focus of current follow-up research in our laboratory. Following the shock therapy with HP, which may take place with or without ongoing nutrient-load reduction measures, the fear for toxic cyanobacterial dominance in lake phytoplankton may be relieved; however, we admit that sustainable reduction of phytoplankton eventually relies on continued reversal of eutrophication in lake ecosystems. Finally, we stress that HP degrades to harmless water and oxygen, and leaves no permanent traces of the added chemical in the lake water. As such the ‘HP method’ appears to be a promising method on its own or serve as an accompanying measure to re-oligotrophication practice to render instant result in lake ecosystem mitigation on demand.

## Author contributions

This study was conceived and designed by HM and EW, conducted by EW and VL, while the laboratory work was supervised by HM and MS, microscopical analysis was performed by VL and MV; EW, HM, and PV wrote the manuscript. All authors declare to agree with the final manuscript and support its submission for publication.

### Conflict of interest statement

The authors declare that the research was conducted in the absence of any commercial or financial relationships that could be construed as a potential conflict of interest.

## Abbreviations

HP: hydrogen peroxide, H_2_O_2_
