# Supplementary material for: Combatting cyanobacteria with hydrogen peroxide: a laboratory study on the consequences for phytoplankton community and diversity
Source: Front Microbiol. 2015 Jul 22;6:714. doi: 10.3389/fmicb.2015.00714 (PMC4510418; doi:10.3389/fmicb.2015.00714)
Supplement: Supplementary file 1 [file Table1.PDF]

SUPPLEMENTARY Table 1

Numbers of cells (cells·mL^{-1}) of different taxa as observed using bright field microscopy. Samples from technical replicates (n=3) of control water (0 mg·L^{-1} HP) and treated water (2.5; 5.0; 10 mg·L^{-1} HP) were collected 25 days after HP addition. Taxa abundance is presented as cell density. Taxa found in samples but not counted in accordance with the method are indicated as zero value.

	**Control (0 mg·L^{-1} HP)**			**Treated (2.5 mg·L^{-1} HP)**			**Treated (5.0 mg·L^{-1} HP)**			**Treated (10 mg·L^{-1} HP)**		
TAXA	**1**	**2**	**3**	**1**	**2**	**3**	**1**	**2**	**3**	**1**	**2**	**3**
Planktothrix agardhii	1,821,818	5,620,364	1,774,545			1,287,273	1,381,818	942,364		507,727	82,727	0
CYANOBACTERIA-TOTAL	***1,821,818***	***5,620,364***	***1,774,545***			***1,287,273***	***1,381,818***	***942,364***		***507,727***	***82,727***	
***Actinastrum* sp.**											727	
Chlorophyta > 5 μm cell	1,0526	8,525	15,738	303,448	196,491	11,803	214,035	1,400,000	276,316	1,035,714	771,429	1,603,448
Chlorophyta > 5 μm colony	3,509		2,623	14,035	77,193	9,180	108,772	14,035		61,404	102,615	0
Chlorophyta 2-5 μm cell	1,754		8,525	89,655	17,544	3,934	143,860	428,571	26,316	285,714	200,000	61,404
Chlorophyta 2-5 μm colony								28,070				
***Closterium* sp.**											91	
Closterium acutum			727	2,182	7,273	13,091	2,000	5,818		1,591	1,636	0
Closterium limneticum			364	636	727	364	364	545	0	455	364	0
***Coelastrum* sp.**						5,246						
Crucigeniella apiculata		0	2,623	28,070			0	28,070			39,722	
***Desmodesmus* sp.**	39,474	20,984	6,0328	1,471,429	365,517	43,934	441,379	1,257,143	333,333	1,785,714	1,057,143	1,706,897
Desmodesmus subspicatus	2,632										6,620	
***Dictyosphaerium* sp.**					203,509				109,649	52,632	211,850	
***Gloeotila* sp.**											9,930	
***Koliella* sp.**										8,772		
Koliella longiseta	0											
***Monoraphidium* sp.**	9,211	2,000	20,364	3,509	0	3,279	7,018	28,070		8,772	9,930	8,772
Monoraphidium contortum							3,509					
Mychonastes jurisii			7,213				35,088	42,105				52,632
***Oocystis* sp.**					3,509		0			35,088		
***Pediastrum* sp.**				727								
Pediastrum boryanum							2,909			3,636	2,909	
Pediastrum duplex		0		2,909				1,455	0	1,818		
***Scenedesmus* sp.**	0	0	5,246	84,211	21,053	1,311	42,105	28,070	17,544	105,263	89,374	87,719
***Tetrastrum* sp.**									17,544			
GREEN ALGAE-TOTAL	***67,105***	***31,508***	***123,750***	***2,000,811***	***892,815***	***92143***	***1,001,038***	***3,261,954***	***780,702***	***3,386,573***	***2,504,342***	***3,520,871***

SUPPLEMENTARY Table 1 (CONTINUED)

Numbers of cells (cells·mL^{-1}) of different taxa as observed using bright field microscopy. Samples from technical replicates (n=3) of control water (0 mg·L^{-1} HP) and treated water (2.5; 5.0; 10 mg·L^{-1} HP) were collected 25 days after HP-addition. Taxa abundance is presented as cell density. Taxa found in samples but not counted in accordance with the method are indicated as zero value.

	**Control (0 mg·L^{-1} HP)**			**Treated (2.5 mg·L^{-1} HP)**			**Treated (5.0 mg·L^{-1} HP)**			**Treated (10 mg·L^{-1} HP)**		
TAXA	**1**	**2**	**3**	**1**	**2**	**3**	**1**	**2**	**3**	**1**	**2**	**3**
Achnanthidium minutissimum	439	4,590	10,492	17,544	3,509	656	0	10,526	8,772			
Aulacoseira granulata				91							91	
***Chaetoceros* sp.**								28,070				
Cyclotella meneghiniana								10,526		8,772	16,551	0
***Cymbella* sp.**				182								
Fragilaria capucina			1,455	818	2,545							
Navicula* sp. *< 25 µm	0				364							
Navicula* sp. *25-50 µm				91								
Nitzschia* sp. *25-50 µm	877	6,545	15,273		23,273	5,818	49,123	63,158	83,333	6,136	43,032	8,772
Nitzschia* sp. *50-100 µm	877	3,636	7,273	4,000	15,273	11,636	10,526	14,035	35,088	2,045	13,241	0
***Ulnaria* sp.**		182	2,545	4,182	1091	2,182	545		0			
DIATOMS-TOTAL	***2,193***	***14,954***	***37,037***	***26,907***	***46,054***	***20,292***	***60,195***	***126,316***	***127,193***	***16,954***	***72,914***	***8,772***
Cryptomonas sp < 15 µm	439		364									
Euglenophyceae < 25 µm						656	0	0	0	682	273	
Euglenophyceae > 25 µm											182	
Phacus tortus								182				
Phacus > 25 µm								0				0
***Trachelomonas* sp.**	439										3,310	
OTHER-TOTAL	***877***		***364***			***656***		***182***		***682***	***3,765***	
