# Supplementary material for: Combatting cyanobacteria with hydrogen peroxide: a laboratory study on the consequences for phytoplankton community and diversity
Source: Front Microbiol. 2015 Jul 22;6:714. doi: 10.3389/fmicb.2015.00714 (PMC4510418; doi:10.3389/fmicb.2015.00714)
Supplement: Supplementary file 2 [file Table2.PDF]

SUPPLEMENTARY Table 2

Biovolume (µm^3·mL^{-1}) of different taxa as calculated, based upon cell counts obtained using bright field microscopy. Samples from technical replicates (n=3) of control water (0 mg·L^{-1} HP) and treated water (2.5; 5.0; 10 mg·L^{-1} HP) were collected 25 days after HP addition. Biovolumes are calculated using the values show in the last column.

	**Control (0 mg·L^{-1} HP)**			**Treated (2.5 mg·L^{-1} HP)**			**Treated (5.0 mg·L^{-1} HP)**			**Treated (10 mg·L^{-1} HP)**			*Biovolume*
TAXA	**1**	**2**	**3**	**1**	**2**	**3**	**1**	**2**	**3**	**1**	**2**	**3**	*µm^3·cell^{-1}*
Planktothrix agardhii	90,726,545	279,894,10 9	88,372,364			64,106,182	68,814,545	46,929,709		25,284,818	4,119,818		49.8
CYANOBACTERIA-TOTAL	***90,726,545***	***279894109***	***88,372,364***			***64,106,182***	***68,814,545***	***46,929,709***		***25,284,818***	***4,119,818***		
***Actinastrum* sp.**											154,545		212.5
Chlorophyta > 5 µm cell	2,273,684	1,841,311	3,399,344	65,544,828	42,442,105	2,549,508	46,231,579	302,400,000	59,684,211	223,714,286	166,628,571	346,344,828	216.0
Chlorophyta > 5 µm colony	757,895		566,557	3,031,579	16,673,684	1,982,951	23,494,737	3,031,579		13,263,158	22,164,846		216.0
Chlorophyta 2-5 µm cell	24,253		117,844	1,239,393	242,526	54,390	1,988,716	5,924,571	363,789	3,949,714	2,764,800	848,842	13.8
Chlorophyta 2-5 µm colony								388,042					13.8
***Closterium* sp.**											45,455		500.0
Closterium acutum			363,636	1,090,909	3,636,364	6,545,455	1,000,000	2,909,091		795,455	818,182		500.0
Closterium limneticum			1,336,636	2,339,114	2,673,273	1,336,636	1,336,636	2,004,955		1,670,795	1,336,636		3675.8
***Coelastrum* sp.**						580,155							110.6
Crucigeniella apiculata			194,329	2,079,663				2,079,663			2,942,920		74.1
***Desmodesmus* sp.**	1,560,422	829,497	2,384,803	58,166,601	14,449,152	1,736,759	17,448,033	49,695,737	13,176,900	70,590,536	41,789,597	67,474,816	39.5
Desmodesmus subspicatus	105,263										264,813		40.0
***Dictyosphaerium* sp.**					13,024,561				7,017,544	3368421	13,558,424		64.0
***Gloeotila* sp.**											309,831		31.2
***Koliella* sp.**										789,474			90.0
Koliella longiseta													198.8
***Monoraphidium* sp.**	671,447	145,800	1,484,509	255,789		239,016	511,579	2,046,316		639,474	723,932	639,474	72.9
Monoraphidium contortum							127,263						36.3
Mychonastes jurisii			99,714				485,053	582,063				727,579	13.8
***Oocystis* sp.**					394,737					3,947,368			112.5
***Pediastrum* sp.**				146,724									201.7
Pediastrum boryanum							327,273			409,091	327,273		112.5
Pediastrum duplex				327,273				163,636		204,545			112.5
***Scenedesmus* sp.**			207,374	3,328,901	832,225	51,844	1,664,451	1,109,634	693,521	4,161,126	3,533,032	3,467,605	39.5
***Tetrastrum* sp.**									242,526				13.8
GREEN ALGAE-TOTAL	***5,392,964***	***2,816,608***	***10,154,748***	***13,755,0774***	***94,368,628***	***15,076,713***	***94,615,319***	***372,335,287***	***81,178,491***	***327,503,443***	***257,362,858***	***419,503,143***	

SUPPLEMENTARY Table 2

Biovolume ($\mu m^3 \cdot mL^{-1}$) of different taxa as calculated, based upon cell counts obtained using bright field microscopy. Samples from technical replicates (n=3) of control water ($0\ mg \cdot L^{-1}$ HP) and treated water (2.5; 5.0; $10\ mg \cdot L^{-1}$ HP) were collected 25 days after HP addition. Biovolumes are calculated using the values shown in the last column.

	Control ($0\ mg \cdot L^{-1}$ HP)			Treated ($2.5\ mg \cdot L^{-1}$ HP)			Treated ($5\ mg \cdot L^{-1}$ HP)			Treated ($10\ mg \cdot L^{-1}$ HP)			*Biovolume*
TAXA	**1**	**2**	**3**	**1**	**2**	**3**	**1**	**2**	**3**	**1**	**2**	**3**	*$\mu m^3\ cell^{-1}$*
Achnanthidium minutissimum	21,380	223,757	511,445	855,213	171,043	31,965		513,128	427,606				48.7
Aulacoseira granulata				66,273							66,273		729.0
***Chaetoceros* sp.**								2768,032					98.6
Cyclotella meneghiniana								16,845,272		14,037,726	26,486,276		1,600.3
***Cymbella* sp.**				21,553									118.5
Fragilaria capucina			481,745	270,982	843,055								331.2
Navicula sp < 25 µm					15,591								42.9
Navicula sp 25-50 µm				58,329									641.6
Nitzschia sp 25-50 µm	189,474	1,413,818	3,298,909		5,026,909	1,256,727	10610526	13,642,105	18,000,000	1,325,455	9,294,935	1,894,737	216.0
Nitzschia sp 50-100 µm	1,515,789	6,283,636	12,567,273	6,912,000	26,391,273	20,107,636	18189474	24,252,632	60,631,579	3,534,545	22,879,841		1,728.0
***Ulnaria* sp.**		632,091	8,849,273	14,538,091	3,792,545	7,585,091	1896273						3,476.5
DIATOMS-TOTAL	***1,726,643***	***8,553,303***	***25,708,645***	***22,722,440***	***36,240,415***	***28,981,420***	***30696273***	***58,021,168***	***79,059,185***	***18,897,726***	***58,727,325***	***1,894,737***	
Cryptomonas* sp. *< 15 µm	663,576		550,165										1,513.0
Euglenophyceae < 25 µm						531,148				552,273	220,909		810.0
Euglenophyceae > 25 µm											336,764		1,852.2
Phacus tortus								574,021					3,157.1
Phacus > 25 µm													2,030.5
***Trachelomonas* sp.**	229,652										1,733,223		523.6
OTHER-TOTAL	***893,228***		***550,165***			***531,148***		***574,021***		***552,273***	***2,290,896***		
