# Supplementary material for: Combatting cyanobacteria with hydrogen peroxide: a laboratory study on the consequences for phytoplankton community and diversity
Source: Front Microbiol. 2015 Jul 22;6:714. doi: 10.3389/fmicb.2015.00714 (PMC4510418; doi:10.3389/fmicb.2015.00714)
Supplement: Supplementary file 3 [file DataSheet1.PDF]

SUPPLEMENTARY MATERIAL (S1)

Phytoplankton species identification by Phyto-PAM

To determine the change in phytoplankton community composition, the relative fluorescence was measured using a Phyto-PAM Phytoplankton Analyzer (Walz, Effeltrich, Germany) instrument. Samples of the various incubations were introduced directly into polystyrene 3 mL four sides-clear fluorescence cuvettes. In this study the differences in biomass prevalence appear as differences in fluorescence output intensity. The Phyto-PAM assay was used just before and four days after HP addition to discriminate between the main phytoplankton groups. The instrument is equipped with a Spherical Micro Quantum Sensor (US-SQS) and measures fluorescence emission after excitation at wavelengths of 665, 645, 520 and 470 nm, and transforms the emitted fluorescence signals into the approximate presence of three differently pigmented algal groups: cyanobacteria, green algae and diatoms/dinoflagellates. Prior to the experiments, excitation spectra of pure cultures of the cyanobacterium *Microcystis aeruginosa* (strain PCC7806), the green alga *Chlorella pyrenoidosa* and the diatom *Phaeodactylum tricornutum* were set as internal reference.

The system operates by means of a computer (software: PhytoWin V2.11, J. Kolbowski, 2009). Cuvettes filled with 3 mL homogenized sample were dark-adapted for 2 min and mounted in the Optical Unit (ED-101US/MP). With actinic light switched off, the measuring light was switched on with a Measuring Frequency Setting 128 (MF128). When the noise level was sufficiently low, indicated by a green LED, the fluorescence signals of the four excitation wavelengths were measured (Figure 1A) and transformed into fluorescence signals for the three algal groups (Figure 1B). The height of a certain fluorescence signal corresponds to an amount of photons emitted by algal pigments and therefore to an amount of a certain algal (pigment) group. In this example, our pure reference cultures are depicted hence values are 100% (Figure 1B).

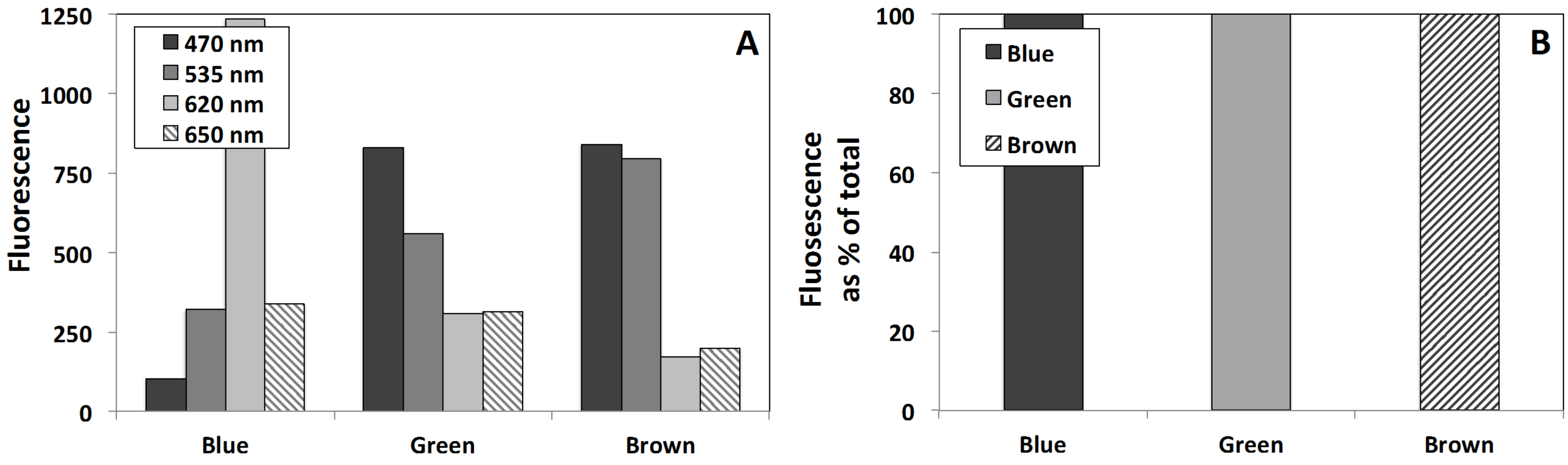

Figure 1. Fluorescence signals measured by the Phyto-PAM instrument at four excitation wavelengths for reference cultures of *Microcystis aeruginosa* (Blue), *Chlorella pyrenoidosa* (Green) and *Phaeodactylum tricornutum* (Brown) (A), and emission values were translated into algal groups (B). These reference signals were used to calibrate the Phyto-PAM. Cuvettes, filled with 3 mL solution consisting of 2 mL growth medium (BG-11) and 1 mL cell culture were measured at a gain of 6, except for pure *Chlorella* which was set at 4. To correct for density differences between cultures, the mean of the four fluorescence signals was set at 500 and corresponding fluorescence values were calculated accordingly. This has no influence on the distribution of fluorescence at different wavelengths.

An example of cultures with more than one phytoplankton group is shown in Figure 2. Again, the Phyto-PAM measures fluorescence emission after excitation at four wavelengths (Figure 2A) and, based upon pure culture-calibration, translated into three algal groups (Figure 2B). For mixing, equal volumes of cultures were used (1:1:1). Since culture densities or biovolumes of the cultures were not perfectly equal, fluorescence emission does not show a similar (1:1:1) distribution.

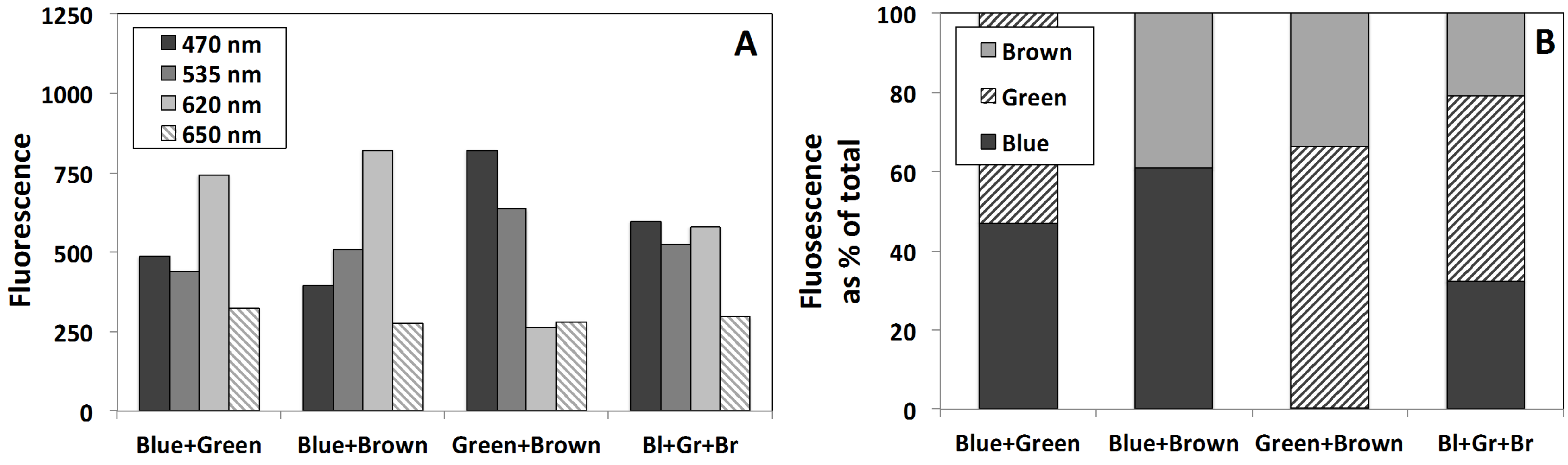

Figure 2. Fluorescence signals mesaured by the Phyto-PAM instrument at four excitation wavelengths for four mixed cultures, based upon the reference cultures. Mixing ratios were 1:1:1 of culture volume (A) and emission values were translated into algal groups (B). Cuvettes filled with 3 mL solution consisting of 2 mL growth medium (BG-11) and 1 mL cell culture (mix) were measured at a gain of 6, except for pure *Chlorella* which was set at 4. To correct for density differences between mix cultures, the mean of the four fluorescence signals was set at 500 and corresponding fluorescence values were calculated accordingly. This has no influence on the fluorescence distribution at different wavelengths nor does it influence translation into algal groups.
